# Diagnostics, correction and prophylaxis of prepathological states using the non-invasive cell technology is one of the ways of longevity

**DOI:** 10.3389/fgene.2015.00184

**Published:** 2015-05-15

**Authors:** Lyudmila P. Sycheva

**Affiliations:** Lab of Genetic Monitoring, Department of Human Ecology, Ministry of Health of the Russian Federation, A.N. Sysin Research Institute of Human Ecology and Environmental HealthMoscow, Russia

**Keywords:** cytogenetic status, multiorgan karyological test, micronucleus, binucleated cells, apoptosis

Chronic diseases (cardiovascular, cancer and others) cause the life quality deterioration and early aging. The development of early preclinical human pathology detection methods is necessary for implementation of the modern “4P medicine” paradigm—Predictive, Personalized, Preventive, and Participatory.

A non-invasive multiorgan karyological test for early diagnostics of health state was developed in a laboratory of genetic monitoring of the Research Institute of Human Ecology and Environmental Health named after A. N. Sysin (Moscow). The method involves microscopic studies of buccal, nasal, urothelial and/or bronchial epithelial cells which includes the entire spectrum of cells nuclei states morphological evaluation. In 1992, Tolbert et al. proposed a method of evaluation of nuclear abnormalities in the human buccal cells. The International Project Human Micronucleus Assay in exfoliated cells (HUMNxl) was initiated in 2007 (www.humn.org; Bolognesi et al., 2013). We proposed to increase the number of investigated biomarkers, tissues and integral indicators. We have also determined the indicative guideline values. We propose to consider the following biomarkers: cytogenetic biomarkers (number of micronuclei, nuclear buds and additionally—atypical nuclei); biomarkers of proliferation (binucleated cells and cells with double nuclei) and biomarkers of cell death, which include cells with early destruction of nucleus (with perinuclear vacuoles, nucleus membrane damage, chromatin condensation and vacuolization, early karyolysis) as well as cells with late destruction of nucleus (pycnosis, karyorrhexis, and late karyolysis). The determination and categorization criteria for diagnostic signs of the entire spectrum of karyological biomarkers was described earlier (Sycheva, 2007). Considering the karyological biomarkers categorization, including of cytogenetic parameters (cyt), proliferation (pr) and destruction of nucleus (apop), an accumulative cytogenetic damage index formula [I_ac_ = (I_cyt_ × I_pr_/I_apop_) × 100] as well as three levels of cytogenetic stress (low, acceptable, or high) were proposed (Sycheva, 2012).

Exfoliative cells are renewed during 10–14 days, so cytogenetic status can be monitored and corrected. It is recommended carrying out analysis of cytogenetic status 2 times in year. If the level of cytogenetic stress is high, it is necessary to determine factors inducing the DNA damage by means of interviewing a patient (eating habits, medication, working conditions, living conditions, social habits, solarium usage, etc.). Taking into account the predominating free radical mechanism of cell damage the vitamin-mineral antioxidant complexes administering may be proposed by recommended daily intakes to eliminate or minimize the identified risk factors. The next evaluation of patient's cytogenetic status is necessary to carry out after 2–3 months to determine the direction of changes. We used this approach to evaluate the influence on the population genetic state of such environmental factors as dioxins, oil-contaminated soils, complex pollution of pulp and paper mill, metallurgical plants, coffee-production enterprise the large office in Moscow etc. Our studies revealed significantly higher numbers of exfoliated cells with cytogenetic damage and, in some cases, the level of apoptosis decreased in the group exposed to environmental pollution, compared to the control group. Decreased apoptosis is very adverse for the population, as it may lead to the accumulation of cytogenetically damaged cells as well as to the cancer induction. Positive results and improvement of human cytogenetic status were obtained after administering of antioxidants. In our study 52% of students have demonstrated decrease frequency of cytogenetically damaged cells after the 1 month of vitamins A and C administration, 38% of students did not have changes, 10% of students have demonstrated higher level of damaged cells within the indicative guideline values range. On the whole the frequency of cells with cytogenetic damage was decreased from 1.93 to 1.17‰ (*P* < 0.05) and apoptotic index was increased from 30.3 to 36.7‰ (*P* < 0.001) (Abasova et al., 2012). Approximately the same results were obtained after the vitamin and mineral complexes ≪ Helvesana ≫, ≪ Lenedex ≫, ≪ Celergen ≫ administering in studies carried out in collaboration with professor Trukhanov et al.

This approach enables the development of “Medicine 4P” paradigm. The “Predictive” medicine concept is based on the analysis of the objective biomarkers of human cytogenetic status and the analysis of epithelial cells which are the most exposed to neoplastic transformation. The “Personalized” medicine means that the evaluation of the cytogenetic status can be carried out for each person and the cytogenetic status monitoring allows determination of deleterious conditions. The “Preventive” medicine concept suggests determination of the earliest deleterious effects before their clinical manifestations. The “Participatory” medicine expects patient participation using non-invasive methods for monitoring their own cytogenetic status at the preclinical stages. The elimination and correction of genotoxic environmental factors may be attempted in cooperation with a patient in order to control and improve their personal cytogenetic status.

## Conflict of interest statement

The author declares that the research was conducted in the absence of any commercial or financial relationships that could be construed as a potential conflict of interest.
